# The Influence of Forest Activities in a University Campus Forest on Student’s Psychological Effects

**DOI:** 10.3390/ijerph18052457

**Published:** 2021-03-02

**Authors:** Jin Gun Kim, Jinyoung Jeon, Won Sop Shin

**Affiliations:** 1 Graduated Department of Forest Therapy, Chungbuk National University, Cheongju 28644, Korea; jingun0308@naver.com (J.G.K.); forest-bb@naver.com (J.J.); 2 Department of Forest Sciences, Chungbuk National University, Cheongju 28644, Korea

**Keywords:** forest therapy, campus forest, profile of mood state, subjective well-being

## Abstract

This study aimed to examine the psychological effects of forest activities in a campus forest. A pre-test and post-test control group design was employed to evaluate the psychological effect of forest activities in a campus forest. A total of 38 participants participated in this study (19 in the forest activities group; 19 in the control group). The Profile of Mood State (POMS) questionnaire, the Concise Measure of Subjective Well-Being (COMOSWB), and the modified form of the Stress Response Inventory (SRI-MF) were administered to each participant to assess psychological effects. This study revealed that participants in the forest activities intervention group had significantly positive increases in their mood, stress response, and subjective well-being, comparing with those of control group participants who did not partake in any forest activities. In conclusion, the implementation of forest activities in a campus forest is an efficient strategy to provide psychological well-being benefits to college students.

## 1. Introduction

The world has become an urban society, with many populations becoming alienated from the traditional people–nature relationship. According to the World Health Organization [1], more than half of the world’s population lived in urban environments in 2014, and this will increase to 65% by 2030. Urbanization, defined as the increase in the number of cities and urban population, is a demographic movement and includes social, economic, and psychological changes that constitute the demographic movement. It is a process that leads to the growth of cities due to industrialization and economic development [2]. Urbanization provides us with many opportunities, such as rapid economic growth, but overexposure to stress causes mental health problems [3,4]. Namely, urbanization affects mental health by increasing stressors and factors such as overcrowding and polluted environment, high levels of violence, and reduced social support [5].

In such a situation, mental health is an increasingly urgent public health problem, highlighted by an increase in mental illness such as depression and anxiety [6,7]. This mental health crisis is a particular concern on college campuses. Students encounter stressors, including academic and extracurricular demands, relationships, financial concerns, familial expectations, identity development, and racial and cultural differences. In recent years, there has been an increase in reported symptoms of mental health in college student populations. According to the 2019 National College Health Assessment [8], 87% of students felt overwhelmed in the past 12 months by all the work they had to do. In addition, 65% of students reported feeling overwhelming anxiety, and 70% of students felt very sad and, 45% of the students suffered from depression. Gallager [9] and Mackean [10] reported that college students had increased mental health problems such as depression, anxiety, suicidal thoughts, and other chronic psychiatric disorders compared to the general population. Research suggests that students have mental problems due to the pressure of academics, taking exams, transitioning into college life [11], relationship problems with a peers and romantic partners [12], financial concerns, and familial expectations [13]. Hence, mental health issues among college students can negatively affect academic performance [14,15], relationship dysfunction [16], a high rate of drinking [17,18] and substance abuse [19,20], and increased incidence of suicide [21]. Therefore, it is important to employ effective interventions for coping with a college student’s mental health problems.

The use of forests and forest therapy is increasingly recognized as an effective intervention for dealing with physical and psychological problems [22,23]. Many studies demonstrated that using forests helps to reduce stress and enhance physiological relaxation [24,25,26,27]. For instance, forest activities, such as walking through a forest or viewing the landscape, can also increase parasympathetic nervous activity [26,27,28], suppress sympathetic nervous activity [29,30], reduce the cortisol concentration [24,31], pulse rate, and blood pressure [28,31], and improve the immune system [32,33,34]. Park et al. [31] reported that the forest walking group demonstrated reduced cortisol levels, blood pressure, heart rate, and sympathetic nervous activity and improved parasympathetic nervous activity compared to the city walking group. Studies by Li et al. [32] investigated the impact of forest trips on natural killer (NK) cell activity for two nights and three days on 12 men with weakened immune function. The results demonstrate that forest activities improve weakened immune function. A further study conducted on women also reported that weakened NK cell activity positively affected male and female participants [33]. In addition, increased NK cell activity was maintained for one week for both men and women, and in the case of men, the long-term effect of forest healing was revealed through the results, in that it was maintained until 1 month later [34].

In addition, regarding psychological relaxation aspects, the effects of forest therapy were associated with improved mood state [35,36], self-esteem [37,38], and quality of life [39,40], decreased psychological stress [41,42], depression and anxiety [43,44,45]. For example, Kaplan and Kaplan [46] argued that individual mental fatigue could be restored through contact with the natural environment, such as the forest. Ulrich et al. [47] demonstrated that natural scenery, such as forests, relieves psychological stress. Shin et al. [48] assessed the impact of forest environment on individual psychological health and well-being and the forest experience contribution to emotional and cognitive health promotion. Regarding the more direct impact of natural environments, such as forests, Pretty et al. [49] reported that participants’ mood and self-esteem improved significantly after forest exercise. Park et al. [50] showed that walking through a forest reduces negative emotions such as tension, anxiety, depression, anger, fatigue, and improves positive emotions such as vigor. In a similar study, Morita et al. [51] reported that staying and walking in the forest reduces hostility and depression and increases vigor, and further study showed that walking in the forest improves the amount of sleep and reduces the anxiety of participants [52]. To further clarify the psychological effects of forests, Song et al. [36] divided 624 college students into 52 groups to walk through the forest and urban environment. The results show that participants who walked in the forest environment experienced decreased negative emotions such as depression, tension, anger, fatigue, and confusion and increased positive emotions such as vigor, compared with participants who walked in the city environment, and participants with high anxiety experienced a greater effect from the forest in improving depression than those with general or low anxiety.

Many studies demonstrated that exposure to nature, such as forests, improves cognitive tasks that require direct attention. For example, Tennessen and Cimprich [53] showed that the student who can see the natural environment through dormitory windows performed better in tasks requiring concentration than those who did not. Taylor et al. [54] also reported that children who could see urban forests near their apartments performed better in tasks relating to memory, impulse control, selective attention, and concentration than those who did not. Similarly, it has been shown that walking in the natural environment, such as forests, provides advantages in language work memory and cognitive control compared with walking in an urban environment [55,56,57].

However, due to the busy college life of students, interaction with forests far away from cities is limited. Therefore, we need to utilize the campus forest to improve students’ quality of life. Campus forests are forests in the university campus space [58]. Campus forests are considered meaningful places for experiencing everyday life, with the potential to support healthy campus planning [59,60]. Campus forests are pre-existing, easily accessible, and effective resources for health [60]. Students who spend most of their time on campus walk by and through these spaces daily. Therefore, it could be a good idea to use campus forests to manage university students’ mental health. Campus forests have recently been placed on the list of important urban green spaces, drawing more attention to them than in the past [61,62]. Campus forests can connect green networks in the inner city and contribute to forming a community with residents. On university campuses, green space is desirable, and represents a precious amenity to help recruit and retain students, promote student loyalty and pride, attract donors, and improve campus quality of life [63].

Some studies suggest the use of campus forests to help college students. Tudorie et al. [64] reported that a campus forest provides a place to relax, meet friends, and pass through. It also has a high potential to provide cultural, provisioning, and regulation landscape services. The content and greenness of the view from indoors on campus can improve indoor settings’ perceived restorativeness [65]. Campus forests also can improve the quality of life and mental health benefits for college students. For example, McFarland et al. [66] showed that college students’ use of campus forests is associated with their quality of life. Hipp et al. [67] also reported that college students who have higher levels of greenness on-campus show a higher quality of life. In addition, Bang et al. [68] also reported that a campus forest walking program significantly increased health-promoting behaviors and parasympathetic nerve activity and decreased depression. Using 558 voluntary college students, Ibes et al. [60] investigated the psychological impact of green micro-breaks on stress reduction. This study revealed that green micro-break interventions provided a significant psychological impact—most commonly, relief from stress. Therefore, utilizing campus forests may serve as an effective strategy to help students to alleviate mental health problems.

Even though a few studies positively evaluated campus forests for alleviating mental health problems, there is still a lack of research in this area. More research evidence is also needed on the health benefits of the use of campus forests. Therefore, this study aimed to investigate whether students’ forest activities influenced their psychological states.

The following research hypotheses were formulated and tested in the study:

(1) Campus forest activities will have a positive effect on mood (on the POMS: The Profile of Mood State scale);
(2) Campus forest activities will have a positive effect on stress response (on the modified form of the Stress Response Inventory: SRI-MF scale);
(3) Campus forest activities will have a positive effect on happiness (on the COMOSWB: Concise Measure of Subjective Well-Being scale).

## 2. Materials and Methods

### 2.1. Participants

Thirty-eight university students (mean age, 22.1 ± 1.6 years) were recruited for the field experiment. The distribution of participants was 24 males (mean age, 22.7 ± 1.4 years; 63.2%) and 14 females (mean age, 21.2 ± 1.4 years; 36.8%). Recruitment posters were posted throughout the university buildings to recruit volunteers. No incentive was provided to the volunteers. The inclusion criteria required the participants to be current students at the specified university. Participants who met the following inclusion and exclusion criteria were considered for study: (1) no diagnosis of a reaction to severe stress and/or depression; (2) did not suffer from drug or alcohol abuse. The participants were randomly distributed into two groups (i.e., 19 in the campus forest activities group and 19 in the control group). The details of participants’ characteristics are shown in Table 1.

The experiment was conducted during the 2nd semester of 2019 (September–November). A total of eight sessions of forest activities were performed. Before starting the experiments, we explained the study’s purpose and procedures and obtained their written informed consent. The Institutional Review Board of Chungbuk National University (IRB number: CBNU-201910-SB-945-01) approved this study.

### 2.2. Experimental Site

The field experiment site was conducted in the Chungbuk National University campus forest in Korea. The size of campus forest is about 315,000 m^2^, covered mainly by *Metasequoia glyptostroboides* (DBH 44cm; tree height 22–27 m; 0.2 ha), *Chamaecyparis pisifera* (DBH 34 cm; tree height 12–18 m; 0.25 ha), and mixed forest species (pitch pine, oak, chestnut, oriental cherry; 2.7 ha), and the stand age ranged between 40 and 90 years old. The study area was a suitable place for conducting forest activities in terms of accessibility, distribution of a variety of vegetation, and gentle slope. During the eight sessions in the experiment, the weather was pleasant and not raining, with a mean temperature of 16.2 ± 1.3 °C.

### 2.3. Procedure

**Figure 1:**
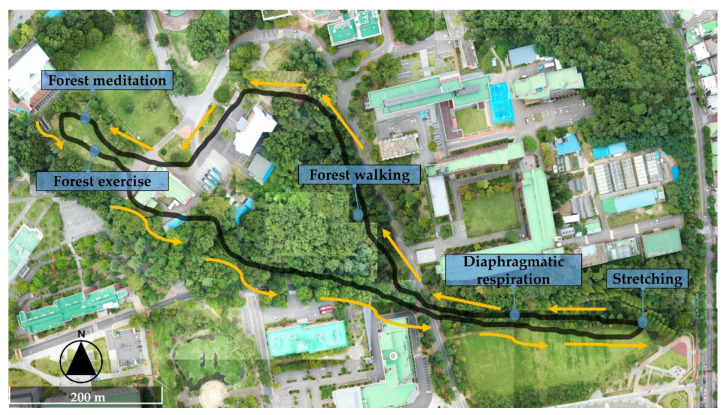
Maps of the campus forest activities trail in this study.

The study employed a pretest-posttest control group experimental design [69]. Participants’ psychological states were measured before and after the intervention. The participants were instructed to perform forest activities for eight sessions (one hour-long session per week). We designed the forest activities to be performed voluntarily (see Figure 1). To perform forest activities, an instruction booklet was administered to each participant. The instruction leaflet was developed based on consultation from experts in the forest therapy field, including researchers and practitioners. Five forest activities were selected in this study to apply as the campus forest activities intervention: stretching, respiration, walking, meditation, and exercise.

**Figure 2:**
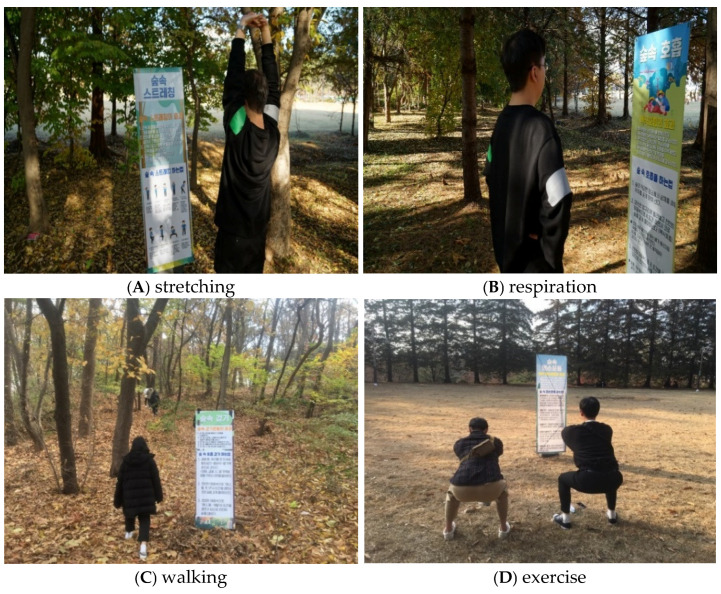

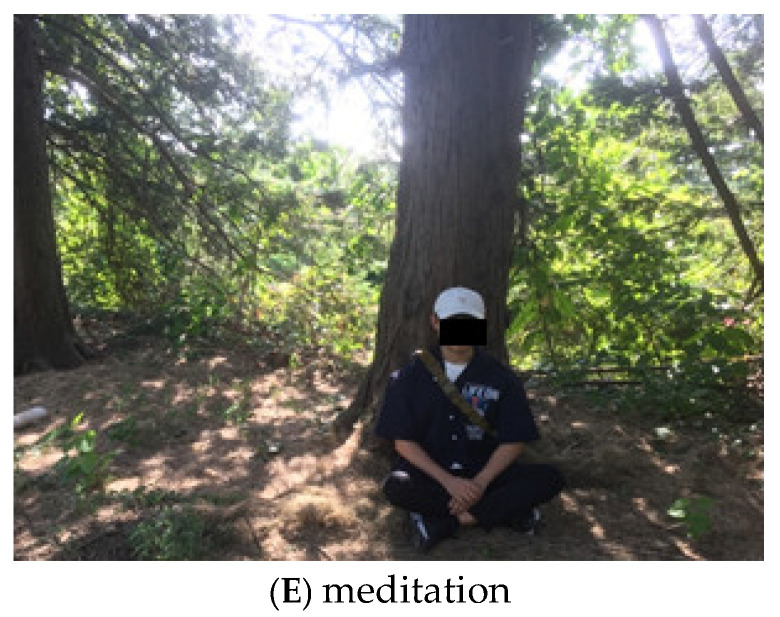
Campus forest activities intervention.

The forest activities interventions were provided from September to November of 2019. The interventions were performed once a week during the daytime, depending on participants’ preferred schedule. During each of the eight sessions in the intervention, participants were asked to walk along the designated route in the campus forest, visit specific locations where explanatory panels on forest activities were installed, and return to the starting point at an appointed time. (see Figure 2). In the first session of the intervention, the leaflet was distributed to each participant. We asked the participants to perform the forest activities intervention at least once a week according to the leaflet’s instructions. Details regarding the instructions used for each activity are included in Appendix A. The participants in the control group did not receive leaflets, lectures, or any forest activities, and were asked to follow out their routine activities during the experimental period. The requests provided to both groups of participants during the intervention are shown in Table 1.

### 2.4. Psychological Measurement

The psychological evaluations used the Profile of Mood States (POMS), the modified form of the Stress Response Inventory (SRI-MF), and the Concise Measure of Subjective Well-being (COMOSWB) questionnaires. The POMS is a well-established measure of emotional state and is a reliable and valid instrument for assessing psychological distress [70,71], and has been used previously to estimate the influence of a forest experience on mood states [71,72,73,74]. It simultaneously evaluates six mood states: “tension-anxiety (T–A)”, “depression-dejection (D)”, “anger-hostility (A–H)”, “fatigue (F)”, “confusion (C)”, and “vigor (V)” [70,71]. A five-point Likert scale (0 = strongly agree to 4 = strongly disagree) was used for each item to evaluate each participant’s mood state. In this study, we employed the short form of the Korean version of the POMS (30 items) [75]. The Korean version of POMS was revealed to have relatively high reliability (Cronbach’s α = 0.85).

The SRI-MF was used to assess the participants’ stress response levels. The SRI-MF is a self-reported tool that measures the participant’s stress response levels, including somatization, anger, and depression [76,77]. The SRI-MF is a key measurement tool concerning stress, particularly the mental health and physical symptoms related to stress [78]. It has been used previously to estimate the effect of a forest therapy program on stress [79]. The SRI-MF has 22 items, and each item has a 5-point Likert scale (1 = strongly disagree; 5 = strongly agree). The SRI-MF was revealed to have relatively high reliability (Cronbach’s *α* = 0.93) [77].

The COMOSWB was used to assess each participant’s subjective well-being states. The COMOSWB measures three sub-scales: life satisfaction, positive emotion, and negative emotion [80]. It has been used previously to estimate the effect of a forest therapy program on happiness [81]. The COMOSWB has a total of nine items with a 7-point Likert scale (1 = strongly disagree; 7 = strongly agree). The COMOSWB was revealed to have relatively high reliability (Cronbach’s α = 0.86) [80].

### 2.5. Data Analysis

The statistical analyses were performed using SPSS 18.0 Windows (SPSS, Chicago, IL, USA). Descriptive statistics comprised means, standard deviation, frequency, and percentage to present socio-demographic information and outcome variables. Paired t-tests were used to compare participants’ psychological states between pre-and post-tests for each group (forest activities intervention and control groups). All statistical tests used a *p*-value of <0.05 as the significance level.

## 3. Results

### 3.1. Psychological Measurements

#### 3.1.1. Profile of Mood States (POMS)

The results of paired t-tests between pre-and post-tests POMS scores for each group are presented in the Table 2. As shown in Table 2, there was a significant decrease in Total Mood Disturbance scores for the campus forest activities group after eight sessions of the forest activities intervention (t = 3.976, *p* < 0.01). The results of paired t-tests indicate that there were significant positive changes in five subscales of the POMS, except for one subscale, entitled “anger-hostility” (t = 1.882, *p* > 0.05): “tension-anxiety” (t = 2.419, *p* < 0.05), “depression-dejection” (t = 2.468, *p* < 0.05), “fatigue-inertia” (t = 5.050, *p* < 0.001), “confusion-bewilderment” (t = 5.175, *p* < 0.001), and “vigor” (t = -2.733, *p* < 0.05).

However, there were no significant changes in the control group participants’ changes in Total Mood Disturbance and all six subscales of the POMS: “tension-anxiety”, “anger-hostility”, “depression-dejection”, “fatigue-inertia”, “confusion-bewilderment”, and “vigor”.

#### 3.1.2. Modified Form of the Stress Response Inventory (SRI-MF)

The results of paired t-tests between pre-and post-tests SRI-MF scores for each group are presented in the Table 3. As shown in Table 3, there was a significant decrease in total stress response scores for the campus forest activities group after eight sessions of the campus forest activities intervention (t = 3.745, *p* < 0.01). The results of paired t-tests indicate that there were significant positive changes in three subscales of the SRI-MF: “somatization” (t = 3.481, *p* < 0.01), “anger” (t = 2.645, *p* < 0.05), and “depression” (t = 2.955, *p* < 0.01).

However, there were no significant changes in the control group participants’ total stress responses and other sub-scales of the SRI-MF: “somatization”, “anger”, and “depression”.

#### 3.1.3. Concise Measure of Subjective Well-Being (COMOSWB)

The results of paired t-tests between pre-and post-tests COMOSWB scores for each group are presented in the Table 4. As shown in Table 4, there was a significant increase in total happiness scores for the campus forest activities group after eight sessions of the campus forest activities intervention (t = −3.202, *p* < 0.01). The results of paired t-tests indicate that there were significant positive changes in two subscales of the COMOSWB: “life satisfaction” (t = −2.996, *p* < 0.01), “negative emotion” (t = 2.750, *p* < 0.05). However, there were no significant changes in the subscale titled “positive emotion.”

On the other hand, there were no significant changes in the control group participants’ total happiness and other sub-scales of the COMOSWB: “life satisfaction”, “positive emotion”, and “negative emotion”.

## 4. Discussion

This study evaluated the psychological effectiveness of forest activities in a campus forest. This study revealed that campus forest activities intervention participants had significantly positive mood states, stress responses, and happiness after the intervention. The results of this study indicate that the campus forest provides effectiveness in improving college students’ psychological health.

To date, many empirical research results have shown that forest therapy programs carried out in forests far away from the city provide a wide range of psychological health benefits to the program participants [82,83]. However, few studies on the psychological effects of campus forest activities have been reported. Therefore, this study suggests that campus forests can help to promote psychological well-being among university students.

This study showed that the negative mood states of “tension-anxiety”, “depression-dejection”, “fatigue”, and “confusion” were lower, and the positive mood states of vigor were significantly higher after campus forest activities intervention. These results, which demonstrate the psychological benefits of campus forest activities, are consistent with previous findings demonstrating improvements in mood states after walking through urban green space [84,85,86] and forest areas [87,88].

This result also found that the total stress responses and other subscales of the SRI-MF such as “somatization”, “anger”, and “depression” were significantly lower after the campus forest activities. This study confirms that campus forest activities had a significant effect on stress reduction. This is consistent with the results of previous studies showing a reduction in psychological stress after viewing a forest and forest walking [88,89,90]. For example, Morita et al. [89] reported that forest walking could improve self-rated health status and reduce psychological stress. Marselle et al. [90] showed that nature-based walking reduced perceived stress, depression, and negative emotion and improved positive emotions and mental health. Im et al. [78] reported that forest walking significantly decreased stress response scores compared to city walking. In addition, Khianman et al. [91] demonstrated the stress-reduction effect of forest therapy on various subjects. These findings show that the benefits of forest therapy activities in stress relief are consistently evaluated as significant results.

This study also shows participants’ increased happiness levels in the campus forest activities intervention group. The results of this study are consistent with previous studies’ improvement of happiness after partaking in forest activities, such as playing in the forest and the forest therapy program. Park et al. [81] reported that employees’ subjective happiness increased after participating in four sessions of a forest therapy program. Happiness increased in children who played in forests comparing with those who played in classrooms [92,93]. Similar studies have shown that green spaces, including forests, promote a sense of happiness [94]. For example, Luck et al. [95] reported that vegetation cover had the strongest positive relation with personal well-being. According to Lohr and Pearson-Mims [96], people felt more positive emotions and happiness when viewing trees than inanimate objects. Van Herzele and De Vries [97] and MacKerron and Mourato [94] reported that people living adjacent to green space were happier than people living in urban environments. People more related to nature tend to seek more experiences with nature, and benefit from the well-being outcomes from those experiences, such as feelings of positive mood and happiness [98,99]. Promoting participants’ happiness offers significant benefits. Happiness can create pleasant moods, positive emotions, positive attitudes toward oneself and others [100], and is known to be significantly linked to reduced heart disease rate [101,102] and mortality [103,104], health and longevity [105,106], and improved sleep [107].

The results of this study reveal a positive relationship between students’ forest activities and their psychological effects. However, it is not clear whether the psychological effects resulted from the activities the participants performed, the forest activities, or exposure to nature and the forest. Further studies are needed to clarify this relationship. The influence of each forest activity performed by the participants on the psychological effects should also be investigated in further research. In particular, forest walking is one of the most representative activities of forest therapy.

Numerous studies have shown that walking in a forest improves mood state [87,88] and alleviates perceived stress [89,90]. Walking in the forest has also been demonstrated to be more effective in improving mental health than walking in the city environment [108]. Diaphragmatic breathing [109,110] and meditation [111,112,113] in the forest have also reported positive psychological effects in some previous studies. Meditation with deep breathing in the forest increases the connection with nature and improves mental, physical, and emotional well-being [114]. The health promotion effects of outdoor activities were significantly enhanced when meditation was incorporated into nature activities [115,116]. Holt et al. [117] showed that students who engaged in the use of campus forests in passive ways, such as sitting, studying, or eating were not associated with health and wellbeing benefits, while students who actively engaged in the use of green space, such as being physically active outdoors, were strongly associated with a higher quality of life, better overall mood, and lower perceived stress. As mentioned above, previous research suggests that time spent in nature in energetic activity can promote restoration and reduce stress and improve happiness [87,88,89,90,91,92,93]. Therefore, forest activities’ psychological effects through campus forests can be an efficient strategy to improve mental health.

Recent research showed that college students are constantly exposed to stressors and that the college period is an important time, during which young people adopt lasting healthy lifestyle habits, and is associated with increased chronic disease risk [118,119]. In particular, mental health problems are important. Across all countries combined, 21.2% of college students have major depression disorders and 18.6% have generalized anxiety disorder [120], and they have a higher rate of being diagnosed or treated by a professional for anxiety, depression, panic attacks, and attention deficit hyperactivity disorder (ADHD) [8]. Universities have been increasing their efforts to address this problem in various programs. The programs for students have included initiatives such as mindfulness programs, yoga classes, and pet therapy [121,122,123]. Although a variety of programs has been used to address students’ mental health, the ability of campus forests to help the student is often overlooked. Students, faculty members, and others use campus forests for a walk every day. However, the potential psychological benefits of campus forests are not widely acknowledged [60]. Therefore, this study can demonstrate the psychological health potential and suggest campus forests for health promotion resources to university students and other campus members.

Forest activity in campus forest is an effective and convenient way to escape busy college life and focus only on oneself. Undertaking activities in natural environments reduces stress and anxiety and thus promotes health [124]. Our study suggests promoting opportunities for students to use campus forests and inform ways or activities for students to utilize campus forests to promote mental health. In addition, staff or faculty of the university can use campus forests in leisure activities whenever they can, such as lunchtime or rest time during work, which will help to relieve work stress. In this regard, the university should continue to maintain well-managed campus forests and expand convenient access to paths through natural areas, promoting campus forest or green space use. The university should also support education to increase students’ awareness around existing campus forests and the potential mental health benefits from regular use [117].

However, we acknowledge the several limitations of this study. Firstly, this study employed five forest activities (stretching, respiration, walking, meditation, and exercise). It is not clear which activity contributed the most to the results and how the differential effect of each of the activities affected the participants. Further studies are needed to clarify and compare the various types of forest activities. Secondly, the sample size of this study was small, and the participants for this study were limited to healthy university students in their 20s. To generalize the findings, further studies are needed create a large sample using different groups of the population with different socio-demographic characteristics. Thirdly, this study was conducted in a campus forest to validate the psychological effect of campus forest activities. The potentials of different forests are not all the same and vary based on forest features. The effects according to the various characteristics of the forests (i.e., tree species composition, forest stand, structure, canopy cover, forest element-related) must be examined in the future. Fourth, participants’ prior expectations and experiences with forests may influence the results. Further study is needed considering the extent of exposure to nature in childhood and patterns of visits to forests. Firth, in this study, the control group conducted their usual activities. Some of the control group may use forests for their leisure, and those experiences may influence the results of this study. Therefore, further studies are needed with participants who spend time in forests without giving them any instructions. Sixth, for an overall discussion, verifying the effects of campus forests using other psychological methods is necessary to demonstrate the new possibility to promote students’ health. In a further study, it is necessary to investigate in depth what are the important variables that contribute to the psychological effects of campus forests. These limitations should be considered in future research. Despite these limitations, this study provides a notable strength of the forest activities in a campus forest, which is that they are easily accessible to students to use whenever they can.

## 5. Conclusions

This study showed that the campus forest activities intervention provided significant psychological effects on college students. More specifically, there were significant positive changes in participants’ emotional states, stress responses, and happiness. The study results indicate the effectiveness of campus forest activities, suggesting the complementary use of different types of forest areas in such a modern urbanized society. These findings can provide guidance to decision-makers on the importance of the campus forest management, and policymakers can utilize these results in order to enhance student’s mental health.

## Figures and Tables

**Table 1:** Instructions given to the two groups of participants during the study period.

Group	Direct Request
Activity group	Read and follow the instructions on the guide signs for each activity installed on the course. Do individual activities, not group activities, when performing forest activities Keep normal life during the experiment Avoid alcohol and tobacco during the experiment Do forest activities regularly (At least once a week)
Control group	Keep normal life during the experiment Avoid alcohol and tobacco during the experiment

**Table 2:** The results of paired t-test analyses of subjective Profile of Mood State (POMS) scores.

	CFA				Cont.			
	Before	After	t	*p*	Before	After	t	*p*
TMD	25.16 ± 4.32	10.68 ± 4.35	3.976	0.001 **	20.79 ± 3.12	21.84 ± 3.85	−0.355	0.726
T–A	7.11 ± 0.96	4.68 ± 1.12	2.419	0.026 *	5.95 ± 0.84	6.42 ± 0.87	−0.590	0.563
A–H	4.68 ± 0.93	3.11 ± 0.62	1.882	0.076	5.79 ± 0.90	5.37 ± 0.92	0.671	0.511
D	5.42 ± 1.04	3.16 ± 0.64	2.468	0.024 *	4.26 ± 0.68	4.58 ± 0.74	−0.480	0.637
F	8.95 ± 0.86	5.42 ± 0.88	5.050	0.000 ***	8.16 ± 0.53	8.32 ± 0.77	−0.221	0.828
C	8.37 ± 0.67	5.74 ± 0.70	5.175	0.000 ***	7.00 ± 0.54	7.11 ± 0.60	−0.244	0.810
V	9.37 ± 0.72	11.42 ± 0.85	-2.733	0.014 *	10.37 ± 0.60	9.95 ± 0.76	0.522	0.608

Notes: T–A, tension-anxiety; A–H, anger-hostility; anger-hostility; D, depression; F, fatigue; C, confusion; V, vigor; TMD, Total Mood Disturbance. Campus forest activities group (CFA) and control group (Cont.). *** *p* < 0.001, ** *p* < 0.01, * *p* < 0.05.

**Table 3:** The results of paired *t*-test analysis of modified form of the Stress Response Inventory (SRI-MF) scores.

	CFA				Cont.			
	Before	After	t	*p*	Before	After	t	*p*
Total	54.16 ± 3.35	43.53 ± 3.34	3.745	0.001 **	49.42 ± 2.90	52.05 ± 3.90	−0.795	0.437
Somatization	22.05 ± 1.46	17.11 ± 1.44	3.481	0.003 **	21.47 ± 1.37	23.74 ± 2.07	−1.320	0.203
Anger	13.84 ± 1.01	11.53 ± 1.04	2.645	0.016 *	10.89 ± 0.78	11.58 ± 0.88	−0.646	0.527
Depression	18.26 ± 1.27	14.89 ± 1.17	2.955	0.008 **	17.05 ± 1.19	16.74 ± 1.33	0.317	0.755

Notes: Total, Total happiness. Campus Forest activities group (CFA) and Control group (Cont.). ** *p* < 0.01, * *p* < 0.05.

**Table 4:** The results of paired *t*-test analyses of Concise Measure of Subjective Wellbeing (COMOSWB) scores.

	CFA				Cont.			
	Before	After	t	*p*	Before	After	t	*p*
Total happiness	19.05 ± 1.61	23.21 ± 1.87	−3.202	0.005 **	21.42 ± 1.65	24.00 ± 1.23	−1.784	0.091
Life satisfaction	14.74 ± 0.68	16.26 ± 0.64	−2.996	0.008 **	16.05 ± 0.60	16.79 ± 0.48	−1.099	0.286
Positive emotion	15.16 ± 0.75	15.63 ± 0.72	−1.027	0.318	14.84 ± 0.80	15.95 ± 0.49	−1.293	0.212
Negative emotion	10.84 ± 0.82	8.68 ± 0.93	2.750	0.013 *	9.47 ± 0.78	8.74 ± 0.81	0.915	0.372

Notes: Campus Forest activities group (CFA) and Control group (Cont.). ** *p* < 0.01, * *p* < 0.05.

## Data Availability

The data presented in this study are available on request from the corresponding author. The data are not publicly available due to privacy.

## Appendix A. Campus Forest Activities Signs Text

(1) Forest stretching signs
  (1) Neck rotation: Stand upright with both hands on your waist and turn your neck in one direction for eight beats. Turn it in the opposite direction.
  (2) Shoulder rotation: Stand upright with your fingertips on your shoulders and turn your shoulders in a circle. Turn it in the opposite direction.
  (3) Waist rotation: Stand upright with both hands on your waist and turn your waist in one direction. Turn it in the opposite direction.
  (4) Knees rotation: Bend your upper body, place your hands on your knees, and turn your knees in one direction. Turn it in the opposite direction.
  (5) Wrist and ankle rotation: Lightly rotate both wrists for eight beats while rotating one ankle together.
(2) Forest respiration signs
  (1) Stand your upper body in a suitable place in the forest with your spine straightened.
  (2) Put your hands on the sides and slowly count ‘1, 2, 3, 4’ (4 s) in your mind and inhale deeply through your nose.
  (3) Slowly exhale into your mouth, counting eight seconds in your mind.
  (4) Repeat 20 times.
(3) Walking in the Forest signs
  (1) When you walk, walk in the order of heel-sole-toe position with your back straight.
  (2) Slowly inhale deeply through your nose, counting ‘1, 2, 3, 4’ (4 s) in your mind.
  (3) Slowly exhale into your mouth, counting eight seconds.
(4) Forest exercise signs
  (1) Push-ups: Stand facing a suitable tree nearby, put your palms on the tree. Bend your elbows and lower your chest to the tree and push through the palms of your hands to straighten your arms (Repeat 10 to 20 times).
  (2) Arm pull: Stretch your arms forward, pull your arms backward as hard as possible, and then stretch them forward again (Repeat 30 times).
  (3) Squat: First, stand your feet shoulder-width apart. Second, squat down by pushing your knees to the side while moving your hips back. Third, break parallel by Squatting down until your hips are lower than your knees. Finally, press into your heels and straighten your legs to return to an upright standing position (Repeat 20 times).
  (4) Core exercise: Keep your knees straight and raise your legs as high as possible (Repeat 10 times on both sides).
  (5) Heel raise exercise: Keeping your knees straight, raise your heels up off the floor as far as you can go, and then slowly return (Repeat 30 times).
(5) Forest meditation signs
  (1) Sit in a comfortable position with your spine straightened by standing your upper body in a suitable place in the forest.
  (2) Slowly inhale deeply through your nose, counting ‘1, 2, 3, 4’ (4 s) in your mind.
  (3) Slowly exhale into your mouth, counting eight seconds in your mind.
  (4) Now, I feel my thoughts and emotions calm as I find the breathing rhythm and focus on breathing.
  (5) If you have miscellaneous thoughts, focus on your breathing.
  (6) At this moment, focus fully on the present state.
  (7) Close your eyes.
  (8) Do it for 5 min.
